# High-Throughput Growth Prediction for *Lactuca sativa* L. Seedlings Using Chlorophyll Fluorescence in a Plant Factory with Artificial Lighting

**DOI:** 10.3389/fpls.2016.00394

**Published:** 2016-03-31

**Authors:** Shogo Moriyuki, Hirokazu Fukuda

**Affiliations:** ^1^Department of Mechanical Engineering, Graduate School of Engineering, Osaka Prefecture UniversityOsaka, Japan; ^2^Japan Science and Technology Agency, PRESTOSaitama, Japan

**Keywords:** circadian clock, chlorophyll fluorescence, diagnosis system, imaging, lettuce, machine learning

## Abstract

Poorly grown plants that result from differences in individuals lead to large profit losses for plant factories that use large electric power sources for cultivation. Thus, identifying and culling the low-grade plants at an early stage, using so-called seedlings diagnosis technology, plays an important role in avoiding large losses in plant factories. In this study, we developed a high-throughput diagnosis system using the measurement of chlorophyll fluorescence (CF) in a commercial large-scale plant factory, which produces about 5000 lettuce plants every day. At an early stage (6 days after sowing), a CF image of 7200 seedlings was captured every 4 h on the final greening day by a high-sensitivity CCD camera and an automatic transferring machine, and biological indices were extracted. Using machine learning, plant growth can be predicted with a high degree of accuracy based on biological indices including leaf size, amount of CF, and circadian rhythms in CF. Growth prediction was improved by addition of temporal information on CF. The present data also provide new insights into the relationships between growth and temporal information regulated by the inherent biological clock.

## Introduction

A plant factory using artificial light offers the potential of stably producing vegetables under constant cultivation year-round, and production can be increased by using vertical multi-cultivating racks (Kozai et al., 2015). However, this approach is more costly than production of outdoor-grown vegetables under sunlight, because the initial costs, and running costs of the equipment are higher. To reduce these costs, reduction of energy costs, development of more efficient environmental control systems, and more effective cultivation protocols are required. Thus, these plant factories require precise environmental control (Morimoto et al., 1995; Kozai et al., 2015). Recently, Li et al. (2016) and Murase et al. (2015) examined the effect of light quality on plant growth. Moreover, Okamura et al. (2014) investigated the optimal harvesting time for vaccine-producing transgenic lettuce and Takahashi et al. (2012) assessed the effect of air flow on production of a vaccine protein against swine edema disease in transgenic lettuce.

Poorly grown plants that do not meet the quality required for sale cause serious losses, reducing the profit of plant factories (Kozai et al., 2015). Poor growth inevitably occurs due to individual differences, even when the same varieties and seeds are cultivated. Thus, identifying and culling low-grade plants at an early stage, using so-called seedling diagnosis technology, is an important process for making plant factories profitable. This technology predicts growth using biological information from seedlings and disposes of seedlings that are predicted to grow poorly (Fukuda et al., 2011).

In large-scale plant factories, statistical values for biological information are stable (Ninness, 2000), because the statistical population of plants is over 1000 every day. Therefore, the accuracy of growth predictors has improved as automatic data acquisition systems and databases to store the biological data have been constructed. In general, multiple visual inspections of leaf size, color, and shape of every seedling provide indices for the assessment of plant growth in commercial factories.

Recently, imaging of chlorophyll fluorescence (CF) has been used as a highly efficient means of visually inspecting plants to assess photosynthetic capacity and degree of stress (Takayama et al., 2014). CF is due to the emission of red light from chlorophyll α pigments (Krause and Weis, 1991; Govindjee, 1995) when residual light energy is not used for photosynthetic reactions. Accurate measurement of CF emission thus allows the evaluation of photosynthetic functions, both the photosynthetic photochemical reactions and the status of heat dissipation processes, without any need for physical contact with the plant (Maxwell and Johnson, 2000; Takayama and Nishina, 2009). The technique of imaging CF, originally developed by Omasa et al. (1987) and Daley et al. (1989), has been used to evaluate the heterogeneous distribution of photosynthetic activities over a leaf surface and thus to detect photosynthetic dysfunctions caused by biotic and abiotic stress factors. Recently, CF imaging has been scaled up to a whole plant (Takayama et al., 2010), a tree canopy (Nichol et al., 2012), and tomato crops cultivated in a large-scale greenhouse (Takayama et al., 2011a,b,c).

CF also exhibits an inherent circadian rhythm, resulting from the regulation of expression of photosynthesis-associated genes by the circadian clock with an approximately 24 h period, and Gould et al. (2009) measured circadian rhythm by the CF in *Arabidopsis thaliana*. Clock genes, which generate circadian rhythms, also regulate growth (Dodd et al., 2005, 2015; Harmer, 2009; Farré and Weise, 2012; Higashi et al., 2014, 2015; Voß et al., 2015). In a previous study of seedling diagnosis, we verified the effectiveness of growth prediction based on the circadian rhythm using a bioluminescent reporter gene assay for transgenic *A. thaliana* carrying the *CCA1*::*LUC* construct, in which the promoter of the *CCA1* clock gene has been fused to a modified firefly luciferase (*LUC*) gene (Fukuda et al., 2011). This study clarified that growth in biomass is correlated with the amount of *CCA1* clock gene expression that was measured via luciferase bioluminescence under various light conditions. In addition, we have investigated features of the circadian rhythm in lettuce cultivars using a similar luciferase-based bioluminescent assay to that of *AtCCA1*::*LUC* (Ukai et al., 2012; Higashi et al., 2014) and evaluated the growth rate of lettuce plants when the circadian rhythm is regulated by conditions of a non-24 h period (Higashi et al., 2015). We speculated that measurement of circadian rhythms will lead to improved plant growth prediction.

Hence, CF monitoring has a practical advantage for simultaneous capture of multiple types of biological information, improving the accuracy of seedling diagnosis. However, there are two tasks needed for construction of a seedling diagnosis system: development of equipment that can measure a time course of CF for a large number of seedlings simultaneously and assessment of the effectiveness of growth prediction based on indices related to the circadian rhythm.

We developed a high-throughput growth prediction methodology for *Lactuca sativa* L. seedlings using CF in a commercial large-scale plant factory, which produces about 5000 lettuce plants every day. The CF of each seedling was measured 6 times every 4 h at 6 days after sowing to detect the circadian rhythm. Multiple types of biological information (six variables including leaf area and the amplitude of the circadian rhythm) were obtained from CF imaging. Finally, we assessed the ability of each variable to predict growth and then combined the variables by machine learning to explore superior indices for seedling diagnosis.

## Materials and methods

### Plant material and growth conditions

Experiments were carried out using lettuce seeds (*L. sativa* L. cv. Frillice and SB555GL, fixed lines of lettuce cultivars from Snow Brand Seed Co., Ltd., Sapporo, Japan).

Two rooms were used for cultivation (rooms A and B); room A was designed for germination and greening of seed cotyledons, and room B was designed for raising seedlings. Room A was equipped with a carrier machine for a greening panel, a white light emitting diode (LED) for greening (LIFELED's; NEC Lighting, Ltd., Tokyo, Japan), and our seedling diagnosis system. Room B was equipped with LED units for raising seedlings (with blue, white, red, and far-red LEDs, GreenPower LED production module DR/W/FR 120, Philips, Amsterdam, Netherlands).

**Figure 1:**
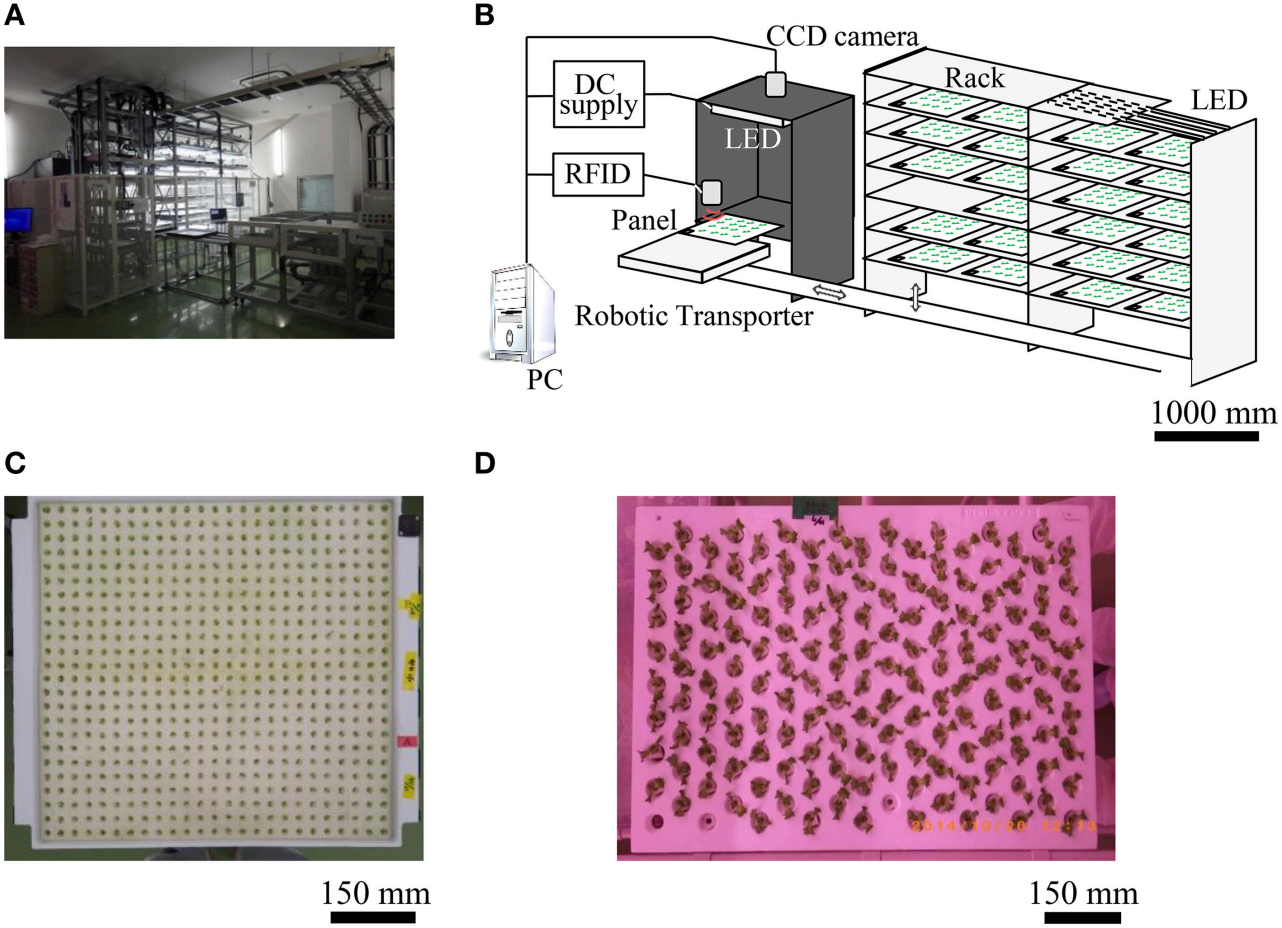
**Seedling diagnostics system and individual panels**. Shown are photographs of **(A)** room A, system chart **(B)**, greening panel **(C)**, and raising panel **(D)**.

In room A, first, each plant was seeded in a greening panel (60 × 60 cm; Figure 1C) allowing 600 plants to be seeded to a urethane sponge sheet (each sponge block was 25 × 25 mm) with 5 L tap water and fertilizer (N:P_2_O_5_: K_2_O:CaO:MgO = 10:8:27:0:4 and N:P_2_O_5_:K_2_O:CaO:MgO = 11:0:0:23:0, Otsuka House No. 1 and 2, respectively; Otsuka Chemical Co., Ltd., Osaka, Japan) at pH 6.0 and EC 0.6. Secondly, the greening panel was laid in the dark at 25°C for 2 days in a growth chamber for germination. Thirdly, plants were cultivated 4 days under white LED light under 15-h light: 9-h dark conditions. Finally, CF of each seedling was measured 6 times every 4 h at 6 days after sowing seeds (see next section). After this measurement, the seedlings were transplanted in a raising panel (60 × 90 cm; Figure 1D) with 153 plantation holes. These seedlings were then raised in room B under 15-h light: 9-h dark conditions at 22°C for 11 days.

To investigate prediction accuracy, we measured fresh weight *W*_*i*_ of 153 Frillice plants and 148 SB555GL plants at 17 days after sowing. We considered the fresh weight of whole plant of Frillice including the sponge and root, whereas fresh weight of SB555GL was weight after removing sponge and root. There was no influence of sponge part: its weight was 0.228 ± 0.002 g.

### Automatic chlorophyll fluorescent measurement system

In a large-scale plant factory, automation is required for seedling diagnosis and transplantation of plants. Thus, we developed a seedling diagnosis system (Figures 1A,B) which can diagnose over 7200 seedlings every day in such a factory. This seedling diagnosis system has a carrier robot for greening panels, a seedling diagnosis apparatus, and a transplanting robot.

The diagnosis apparatus is made up mainly of a dark box (900 mm in width, 900 mm in depth, and 1200 mm in height), a highly sensitive charge coupled device (CCD) camera (Hamamatsu ORCA-Flash4.0; Hamamatsu Photonics KK, Shizuoka, Japan) in the upper dark box, 8 blue LED panels [ λ_*p*_ = 470 nm, 150 × 150 mm at the base; 4 ISL-150X150-HBB blue panels (CCS Inc., Kyoto, Japan), and 4 VBL-SL150 blue panels (Valore Corp., Kyoto, Japan)] in the dark box to excite the chlorophyll of the seedling. In addition, it included a PC-controlled CCD camera, LED controller, RFID system (V680-CA5D02-V2; OMRON Corporation, Kyoto, Japan), digital input/output unit (DIO-6464L-USB; CONTEC Co., Ltd., Osaka, Japan), and automatic acquisition/analysis program for leaf area, CF, and circadian rhythms.

**Figure 2:**
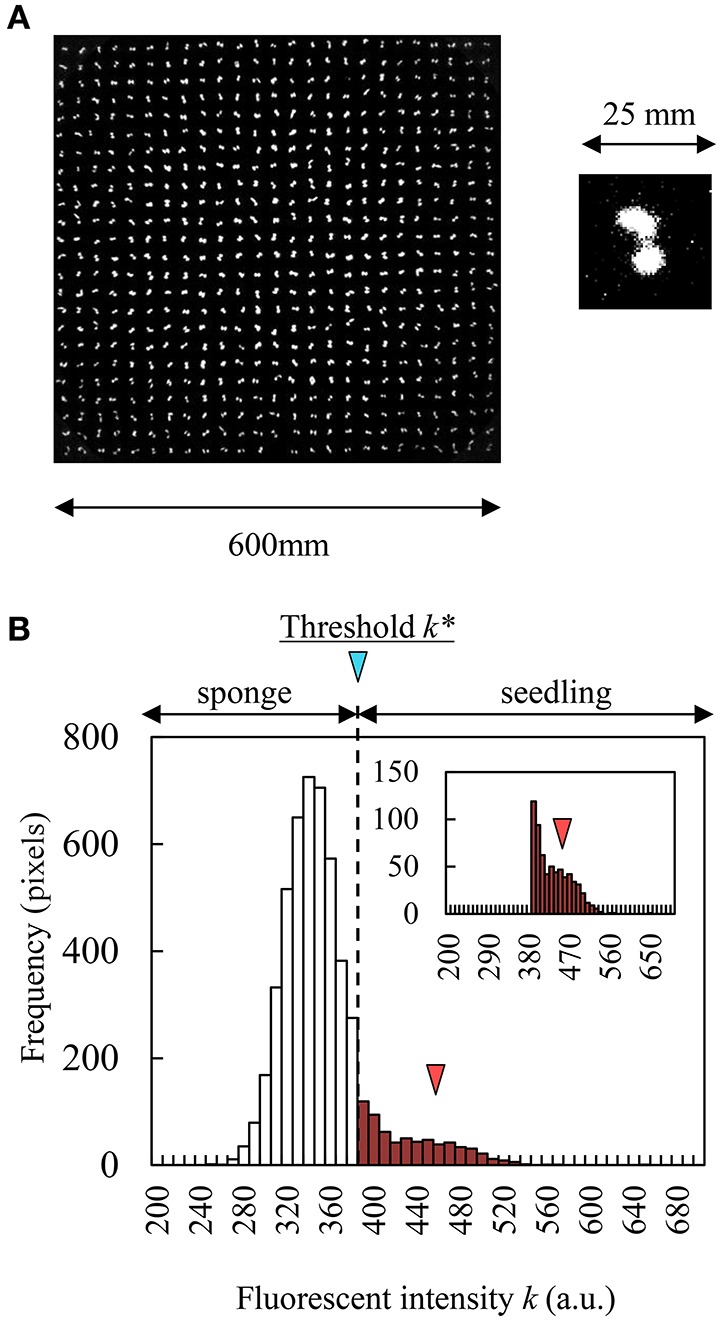
**CF obtained by CCD camera and histogram of fluorescent intensity in a seedling and sponge image. (A)** Grayscale CF image of 600 seedlings (contrast changed). **(B)** Histogram of fluorescent intensity of sponge (white bars) and a seedling (red bar). Red triangle indicates the peak of histogram in a seedling.

In the seedling diagnosis system, at the time of seedling diagnosis on day 6 after sowing, the greening panel carrier robot automatically carried a target panel to the dark box. Immediately, seedlings were illuminated with blue LED light (30 μmol m^−2^ s^−1^) for 2 s to excite chlorophyll. Then, a CF image, such as in Figure 2A, was obtained by CCD camera immediately after the blue LED was turned off. The exposure time of the CCD camera was set to 2 s and the CF image was captured 14 times sequentially for 30 s in one measurement. This measurement was repeated 6 times every 4 h.

We also utilized an RFID system to input seedling diagnosis results, and a digital input/output unit to control opening and closing of the shutter that the dark box was equipped with. Based on the results, we were able to transplant only superior seedlings from the greening panel to the raising panel by a transplant robot automatically.

### Methods of calculating leaf area and circadian rhythms

To calculate leaf area, the grayscale image acquisition CCD camera captures the distribution of fluorescence intensity between sponge and seedling simultaneously. Discriminant analysis is a method to separate the seedling distribution from intensity distribution mechanically (Phan and Cichocki, 2010). Using this method, we could automatically obtain the threshold k^*^ that corresponds to the maximum value of the separation metric *f*(*k*) that compares between-class variance and within-class variance. The separation metric *f*(*k*) is described by:

$$\begin{array}{l} f\left(k^{*}\right)=\underset{0\leq k<L}{\max}f\left(k\right)\\ =\underset{0\leq k<L}{\max}\frac{n_{1}\left(k\right)\cdot {\left(\mu_{1}\left(k\right)-\mu_{0}\right)}^{2}+n_{2}\left(k\right)\cdot {\left(\mu_{2}\left(k\right)-\mu_{0}\right)}^{2}}{n_{1}\left(k\right)\cdot \sigma_{1}^{2}\left(k\right)+n_{2}\left(k\right)\cdot \sigma_{2}^{2}\left(k\right)} \end{array}$$

where *n*_1_(*k*), μ_1_(*k*), and $\sigma_{1}^{2}\left(k\right)$ are the number of pixels, the average, and the variance of fluorescence distribution that was less than *k* (sponge distribution in Figure 2B), respectively. On the other hand, *n*_2_(*k*), μ_2_(*k*), and $\sigma_{2}^{2}\left(k\right)$ are the number of pixels, the average, and the variance of fluorescence distribution that was greater than *k* (seedling distribution in Figure 2B), respectively. μ_0_ is the average of the whole distribution, and *L* is the maximum number of *k* (*L* = 2^16^). If the fluorescence intensity from a pixel was greater than k^*^, this pixel was determined as belonging to the leaf area, and if not, to the sponge block region. We calculated k^*^ in each sponge block region (71 × 72 pixels).

**Figure 3:**
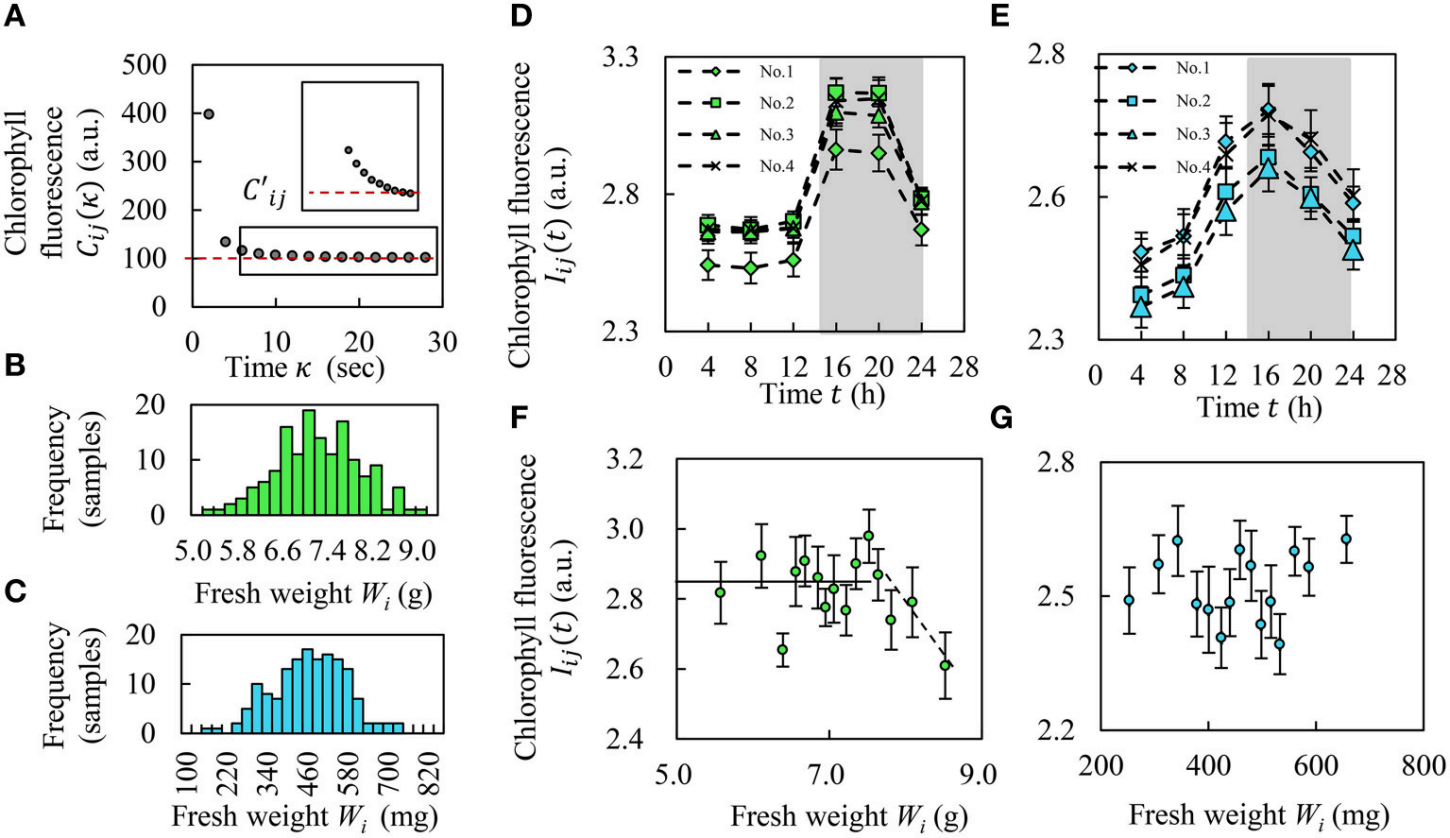
**Amount of chlorophyll and relationship between CF and fresh weight. (A)** Red line shows convergence value, $C_{ij}^{\prime }$. **(B,C)** Histograms of fresh weight in Frillice **(B)** and SB555GL **(C)**. **(D,E)** Alteration of CF *I*_*ij*_(*t*) over the course of a day in Frillice **(D)** and SB555GL **(E)**, averaging *I*_*ij*_(*t*) into four categories (see Results Section). The white and gray background colors indicate light and dark conditions. **(F,G)** Relationship between the average value of fresh weight *W*_*i*_ and the average of CF from each top 10 plants for fresh weight of Frillice **(F)** and SB555GL **(G)**. All error bars mean standard error.

Next, to calculate the circadian rhythm of CF, sequential CF images were captured every 2 s 14 times with a 2 s exposure time to measure the delay curve of CF (Figure 3A); this imaging was performed 6 times every 4 h. For a defined leaf area, we obtained the CF per seedling *C*_*ij*_(κ), where κ means time, *i* means seedling ID, and *j* means the number of measurement times. The CF decreased in time immediately after blue LED turn-off, and the light intensity converged to the constant value *C*′_*ij*_ (Figure 3A). We defined the amount of CF as *I*_*ij*_(*t*) normalized by *C*′_*ij*_ as follows:

$$I_{ij}(t)\;=\int_{0}^{\kappa^{*}}\left(\frac{C_{ij}(\kappa )-C^{\prime }{}_{ij}}{C^{\prime }{}_{ij}}\right)d\kappa$$

where κ = 0 means the time of blue LED turn-off in the dark box, and the constant value *C*′_*ij*_ occurs at κ = κ^*^.

We obtained *I*_*ij*_(*t*) for each seedling 6 times (*n* = 6) every 4 h. We calculated the amplitude *a*_*i*_ and the peak phase φ_*i*_ (0 ≤ φ_*i*_ < 2π) that corresponds to the maximum value of the determination coefficient (Halberg et al., 1972) as follows:

$$\begin{array}{l} y_{i}(t)=a_{i}\cos\left(2\pi \frac{t}{T}-\varphi_{i}\right)+\frac{1}{n}\sum_{j=1}^{n}I_{ij}(t)\\ \;A_{i}=a_{i}/\frac{1}{n}\sum_{j=\;1}^{n}I_{ij}(t) \end{array}$$

In our study, when *t* = 0, the white LED used for greening was turned on in room A. *T* is the light period (in this case, 24 h). In addition, we defined the normalized amplitude *A*_*i*_ as the seedling diagnosis index.

## Results

### Correlation between CF and fresh weight

Figure 3A shows that the CF decreased over time immediately after blue LED turn-off and then converged to a constant value *C*′_*ij*_ of about 30 s. Figures 3B,C show histograms of the fresh weight of Frillice and SB555GL, respectively. Both distributions were nearly Gaussian. Figures 3D,E shows the alteration of *I*_*ij*_(*t*) during a day from one morning to the next. As the average of the fresh weight *W*_*i*_ is μ_*w*_ and its standard variation is σ_*w*_, we separated plants into four categories based on these values: 1 (μ_*w*_ + σ_*w*_ < *W*_*i*_), 2 (μ_*w*_ < *W*_*i*_ ≤ μ_*w*_ + σ_*w*_), 3 (μ_*w*_ − σ_*w*_ < *W*_*i*_ ≤ μ_*w*_), and 4 (*W*_*i*_ ≤ μ_*w*_ − σ_*w*_). From these results, we found that both cultivars strongly emitted CF at night. Moreover, CF decreased with higher weight over a threshold (*W*_*i*_ > 7.6 g) in Frillice; on the other hand, SB555GL did not show such a tendency (Figures 3F,G). The vertical axis in Figures 3F,G mean the average of CF from 10 plants for fresh weight.

### Correlation between indices of circadian rhythms and fresh weight

**Figure 4:**
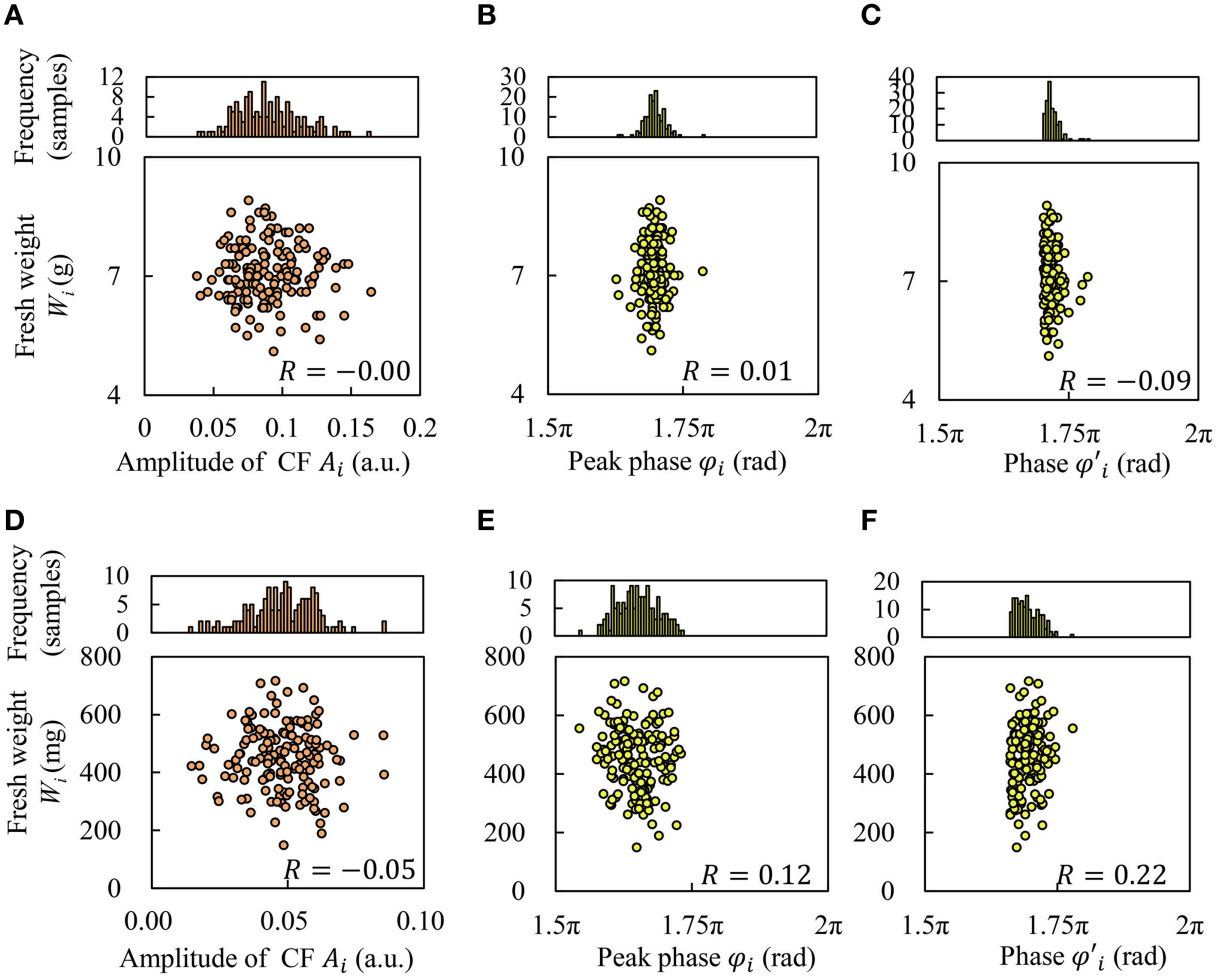
**Correlation between fresh weight and index of circadian rhythms of CF**. Frillice: **(A–C)**; SB555GL: **(D–F)**. **(A,D)** Amplitude *A*_*i*_, **(B,E)** peak phase φ_*i*_, and **(C,F)** phase φ′_*i*_.

Figures 4A,B,D,E show the relationship between *W*_*i*_ and the amplitude *A*_*i*_ and peak phase φ_*i*_. Based on these results, we found that these indices of circadian rhythm did not have any correlation with *W*_*i*_; that is, the correlation coefficient *R* was low. In Figures 4B,E, the center value and range of φ_*i*_ differed depending on the cultivar. Hence, we also defined a baseline ${\bar{\varphi }}_{i}$ for peak phase φ_*i*_, and obtained phase $\varphi_{i}^{\prime }$ to consider the environmental synchrony.

$$\varphi_{i}^{\prime }=\bar{\varphi_{i}}+\left|\bar{\varphi_{i}}-\varphi_{i}\right|$$

$\bar{\varphi_{i}}$ for Frillice and SB555GL was 1.70π rad and 1.66π rad, respectively. Figures 4C,F shows the correlation between phase $\varphi_{i}^{\prime }$ and *W*_*i*_, for which a weak correlation was observed in SB555GL. It seems that growth prediction may be improved by considering the phase of the circadian rhythm.

### Growth prediction using machine learning

**Figure 5:**
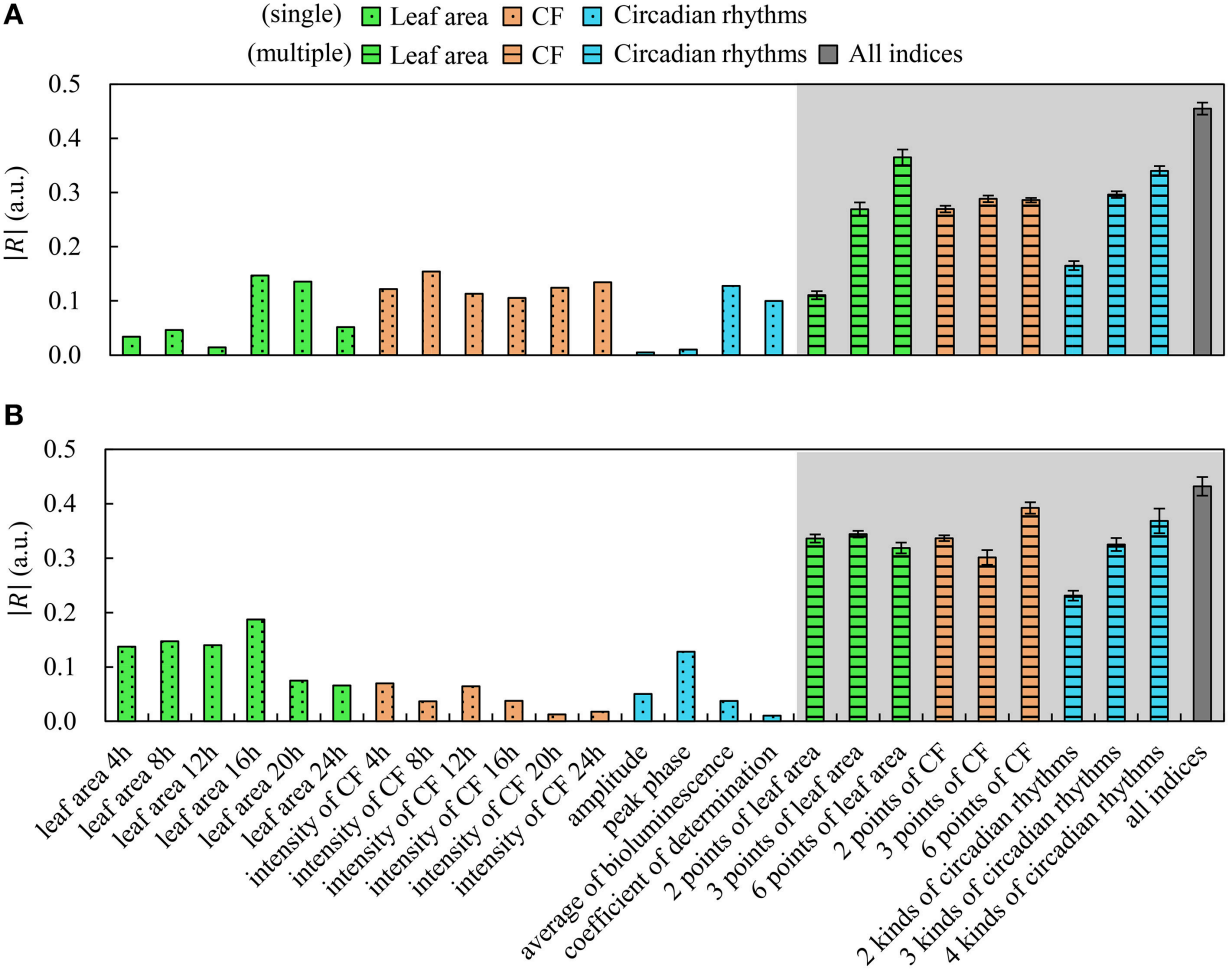
**Correlation between indices and fresh weight using machine learning**. Frillice **(A)**; SB555GL **(B)**. White background indicates a single index, and gray background indicates multiple indices. All error bars indicate standard error.

Neural networks have the inherent capability of learning unknown nonlinear properties (Chen et al., 1990). Morimoto and Hashimoto (2009) used neural networks to control environmental factors in plant factories, and Hendrawan and Murase (2011) used neural networks to predict water content of moss using RGB intensity. In our study, we predicted plant growth using a neural network based on biological information, including 6 time measurements of leaf area and CF (e.g., at 4, 8, 12, 16, 20, and 24 h), and 4 circadian rhythm features. These circadian rhythm features are amplitude *A*_*i*_, peak phase φ_*i*_, average of CF $\left(<I_{i}>=\frac{1}{n}\overset{n}{\underset{j=1}{\sum }}I_{ij}\left(t\right)\right)$, and the determination coefficient of curve approximation *y*_*i*_(*t*). We created a neural network containing up to 16 kinds of biological information in an input layer and *W*_*i*_ in an output layer using 70% of all plant data as training data by a back-propagation method. We used neural networks 40 times with several types of input data, and estimated the average correlation coefficient *R* and standard error. Figure 5 shows the magnitude of the correlation coefficient |*R*| between each index and *W*_*i*_. The white background in Figure 5 indicates a single index; that is, single data points were used for leaf area at each time, CF at each time, amplitude, peak phase, average of CF, and determination coefficient. The gray background in Figure 5 indicates multiple indices: leaf area (2, 3, and 6 points), CF (2, 3, and 6 points), circadian rhythms (2, 3, and 4 kinds), and all biological indices. We defined 2 points of leaf area and CF as meaning data acquired 2 times (at 12 and 24 h) and 3 points of leaf area as meaning data acquired 3 times (at 8, 16, and 24 h). In addition, we defined 2 kinds of circadian rhythms by *A*_*i*_ and φ_*i*_, 3 kinds of circadian rhythms by *A*_*i*_, φ_*i*_, and < *I*_*i*_ >, and 4 kinds of circadian rhythms by all of them plus the determination coefficient. We found that growth was better predicted by multiple indices over a single index.

## Discussion

In this study, we found that the fresh weight of Frillice and SB555GL showed Gaussian distributions based on the Kolmogorov-Smirnov test. Figures 3D,E shows that Frillice and SB555GL have circadian rhythms and a peak of CF in the evening for all plants, supporting the results of Gould et al. (2009). We have succeeded in developing a seedling diagnostics system that automatically measures CF and circadian rhythms of each plant at an early stage simultaneously. Figure 3F shows fresh weight in Frillice decreases with increase of CF, up to a certain threshold based on fresh weight. In other words, when this seedling is under a certain threshold value of CF, its growth is better. This observation suggests that seedlings that use a lot of light energy in photochemical reactions, which would decrease the CF, have higher growth potential.

As can be seen in Figure 4, it would appear that growth prediction can be improved by considering environmental synchrony. It is known that the dry weight increase of *A. thaliana* in a 24 h light cycle is better than in a 20 or 28 h cycle (Dodd et al., 2005; Fukuda et al., 2011), suggesting that the relationship of phase between circadian rhythms and environmental cycles strongly affect plant growth. Thus, we introduced the peak baseline $\varphi_{i}^{\prime }$ to investigate the effect of the relationship of phase between circadian rhythms and environmental cycles. The peak baseline $\varphi_{i}^{\prime }$ was slightly better than the original peak phase φ_*i*_ for growth prediction, as shown in Figure 4F. Moreover, the blue LED light pulses for excitation of chlorophyll would provide no effect on the circadian rhythms. In our previous work (Ohara et al., 2015a,b), it was investigated how plant circadian clock responds to light pulse perturbations. The phase shift of circadian rhythm became maximally to 0.4 rad/2π (~9.6 h) by a blue LED light pulse (80 μmol m^−2^ s^−1^ for 2 h). Based on this knowledge, the phase shift by our diagnostic lighting could be estimated as very small.

As shown in Figure 5, by increasing the number of measurements, the correlation coefficient *R* was improved. An increased number of measurements about leaf area led to improved prediction of growth in Frillice. In contrast, only two measurements of leaf area tended to effectively improve prediction of growth in SB555GL. Therefore, the optimal set of predictive indices depends on cultivar and/or dataset. In addition, using indices of circadian rhythms, no significant difference was observed for combinations of circadian rhythms and leaf area, or for circadian rhythms and CF. For growth prediction using all biological indices, it was significantly different from growth prediction using other indices inferred by machine learning in Frillice; on the other hand, the growth prediction using all biological indices was significantly different from indices other than the 6 time points for CF and the 4 kinds of circadian rhythms in SB555GL. Therefore, it is necessary to decide whether to acquire information on circadian rhythm, and we suggest that the research goal may depend on whether growth prediction can be based on circadian rhythms.

Fukuda et al. (2011) referred to improvement of plant productivity under several LED light conditions by selection of a threshold for an index *I* using the correlation coefficient *R* between production *P* and index *I*. In our study, *I* is the data output by neural networks, and *R* is the correlation coefficient between fresh weight *W*_*i*_ and the data output by neural networks. Therefore, as suggested by Figure 5, improving the value of *R* would lead to improvements in productivity; thus, we expect this seedling diagnosis system will be useful.

In conclusion, we developed a seedling diagnosis system that automatically identifies and selects plants showing poor growth based on biological information obtained at an early stage. We expect that this system will decrease operational cost in plant factories due to individual differences in plants. Using this system, we automatically obtained leaf area, CF, and information on circadian rhythms and suggested improvements to the prediction of growth by machine learning. We found that the system predicted plant growth with a high degree of accuracy; however, the mechanisms of plant growth have yet to be clearly identified. Future research will focus on predicting growth with additional accuracy by the use of environmental information in plant factory.

## Author contributions

HF and SM designed the experiments, and developed a high-throughput diagnosis system. SM performed biological data analysis. SM and HF wrote the manuscript. All authors discussed the results and implications and commented on the manuscript.

### Conflict of interest statement

The authors declare that the research was conducted in the absence of any commercial or financial relationships that could be construed as a potential conflict of interest.
